# Effects of experimental pain on the cervical spine reposition errors

**DOI:** 10.1186/s12891-022-05170-7

**Published:** 2022-03-17

**Authors:** Xu Wang, Ning Qu, Yang Wang, Jian Dong, Jianhang Jiao, Minfei Wu

**Affiliations:** 1 The Department of Spine, The second Hospital of Jilin University, Jilin University, Changchun, 130041 China; 2 The Department of Orthopaedics, The first Affiliated Hospital of Nanchang University, Nanchang, 330006 China; 3 The Department of Rehabilitation, China- Japan Union Hospital of Jilin University, Changchun, 130021 China

**Keywords:** Cervical spine, Experimental pain, Reposition errors, Cervical joint reposition, Spine

## Abstract

**Background:**

Healthy subjects showed normal variance of cervical spine reposition errors of approximately 2 degrees. Effects of experimental pain on cervical spine reposition errors were unknown; thus, the purpose of this study was to investigate the effects of experimental pain on cervical spine reposition errors.

**Methods:**

A repeated measured study design was applied. Thirty healthy subjects (12 males) were recruited. Reposition errors were extracted from upright cervical positions before and after cervical flexion movement in healthy subjects before and during experimental neck pain. Cervical spine reposition errors were calculated based on anatomical landmarks of each cervical joint. Reposition errors were extracted in degrees as constant errors and absolute errors for further statistical analysis. Repeated measures analysis of variance (RM-ANOVA) was applied to analyse experimental pain effects on either constant errors or absolute errors of different cervical joints.

**Results:**

The cervical spine showed non-significant difference in reposition errors regarding the constant errors (*P*>0.05) while larger reposition errors regarding the absolute errors during experimental pain compared to before experimental pain (*P*<0.001). In addition, the pain level joint (C4/C5) and its adjacent joints (C3/C4 and C5/C6) indicated larger reposition errors regarding absolute errors (*P*=0.035, *P*=0.329 and *P*=0.103, respectively).

**Conclusions:**

This study firstly investigated the cervical spine reposition errors in experimental neck pain and further found the joints adjacent to the pain level showed larger errors compared to the distant joints regarding absolute errors. It may imply that the larger reposition errors in specific cervical joint indicate probable injury or pain existed adjacent to the joints.

**Supplementary Information:**

The online version contains supplementary material available at 10.1186/s12891-022-05170-7.

## Background

Cervical upright position is the baseline for diagnosis and studies of cervical disorders, such as alignment measures, vertebral slide measures and proprioception evaluation by repositioning errors [1–5]. Previous studies demonstrated that the variation of cervical spine reposition errors is about 2 degrees in healthy controls regarding cervical spine as multi-joints rather as a whole, which suggests an essential parameter for evaluating if there are disorders in the cervical spine [3]. Cervical reposition errors in healthy controls vary about 2 degrees across multiple cervical joints indicating as a probable baseline for cervical spine disorders when the reposition errors exceeding it. However, it is unknown if the reposition errors in neck pain conditions may exceed 2 degrees or the reposition errors may be distributed across the cervical joints.

Experimental neck pain models induced by different substances provided possibilities investigating pain from different origins in the cervical region within healthy subjects [6–11]. Pain induced in the deep cervical muscle (e.g. multifidus) by hypertonic impaired the proprioception and anatomically influenced the localized neuromuscular control of cervical joints [12].

Impaired proprioception reflecting by increased repositioning errors has been demonstrated in patients with cervical spine disorders [13]. Decreased head reposition accuracy has been showed in patients with cervical radiculopathy [14, 15]. Further, head and neck repositioning accuracy were impaired in other conditions, such as in aging, cervical spondylosis, cervicogenic dizziness, whiplash, muscle fatigue, and non-traumatic neck pain [16–21]. Accordingly, the proprioception has been demonstrated impaired in experimental neck pain subjects by showing increased repositioning errors, while it is still unclear how the errors in specific cervical joints alter and re-distribute. More importantly, the errors in specific cervical joint and the distribution of it may suggest evidence for identifying the probable origin of the cervical disorders. Especially, it might be helpful for origin identification in nonspecific neck problems.

The slender spring-like cervical spine is quite vulnerable to injury during bending [22, 23], thus sensorimotor processes are essential for maintaining head and neck stability and mobility. Cervical sensorimotor function was reflected by muscle function in the cervical spine, of which muscle spindles are also involved in simple stretch reflex, as they are important in controlling head movement and stability and protecting underlying spinal injury [22]. Attenuated sensorimotor function was demonstrated in experimental muscle pain models [24, 25]. Therefore, only cervical flexion movement was performed in the current study for evaluating repositioning ability under experimental pain condition. Moreover, experimental pain models also provide the possibility to investigate pain from different cervical muscles (deep neck muscles) on cervical repositioning errors within subjects [6, 12]. Thus, the purposes of this study are to 1) assess the cervical spine repositioning errors in experimental neck pain conditions and 2) investigate the distribution of repositioning errors of cervical joints for specific level of pain. It was hypothesized that 1) experimental neck pain indicates more reposition errors and 2) the errors of specific joints are larger adjacent to the pain level.

## Methods

### Sample Size

The sample size was calculated based on reposition error variability in healthy subjects in previous study [3]. The effect size of normal variability on healthy cervical joint reposition error parameters at individual cervical joint ranged from 0.16 to 0.62 regarding absolute errors. Considering the experimental neck pain effects on cervical repositioning ability of the cervical spine, the effect size of experimental neck pain on cervical joint reposition error parameters was assumed to larger than normal variability. With enough power to detect significant changes in all flexion motion parameters, the effect size of 0.7 was applied to calculate the sample size. With a significance level of 0.05 and power of 0.9, a minimum of twenty-seven participants was required (G*Power, version 3.1.9.7). Thus, thirty subjects were recruited in this study with a possibility of three subjects drop out due to different reasons.

### Participants

Eighteen pain free females (age: 35.8 ± 4.1 years; height: 169.2 ± 3.6 cm; weight: 60.4 ±5.7 kg; body mass index: 22.5 ± 1.8 kg/m^2^; mean ± standard deviation) and 12 pain free males (age: 37.8 ± 2.1 years; height: 179.2 ± 5.6 cm; weight: 73.4 ±4.3 kg; body mass index: 23.5 ± 2.8 kg/m^2^; mean ± standard deviation) without symptoms in the last half year were included. The study included 30 pain free subjects (12 males) within the last six months (Table 1). Exclusion criteria were possible pregnancy, any disorders of the cervical spine, inability to cooperate. All participants were recruited from university students and volunteers via bulletins and a website.

**Table 1:** General characteristics mean (±SD) of the subjects

Items	Males	Females
Height (cm)	169.2±3.6	179.2±5.6
Weight (kg)	60.4±5.7	73.4±4.3
Age (year)	35.8±4.1	37.8±2.1
BMI (kg/m^2^)	22.5±1.8	23.5±2.8

### Ethics, consent and permissions

The study was conducted strictly according to the Declaration of Helsinki (1968) and approved by the local hospital ethics committee (SB2020249). All details of the experiment were explained to the participants and they signed a written and informed consent form.

### Experimental procedure

The repeated-measures design was applied that the subjects were asked to perform two head and neck repositioning processes separated by five minutes during which experimental neck pain was induced. Each subject was instructed to practice for several times repositioning head and neck to the initial position after flexion before the recording. Then they were asked to return to the upright position after cervical flexion movement before (first repositioning process) and during (second repositioning process) experimental muscle pain. The subjects performed flexion movement and repositioning with their eye open to follow the middle line to reduce the vertebrae distortion in the sagittal plane as it may influence the image quality [3]. The cervical flexion movement was performed whenever the pain intensity was at least 3 on a 10-cm visual analogue scale (VAS) (“no pain” at 0 and “worst pain imaginable” at 10).

### Experimental muscle pain

The hypertonic saline-induced muscle pain model has been applied to study the sensory and motor alterations related to pain in former studies because it mimics clinical acute muscle pain [6]. The experimental muscle pain was induced by injecting a 0.5 ml bolus of sterile hypertonic saline (5.8%) in right cervical multifidus [26]. The right multifidus muscle was injected in the deepest layer at the C4 level, which originates from the articular pillar of C5/C6 junction and inserts on the laminae of C3 [26, 27]. All the injections were guided by ultrasonography [28]. The saline-induced muscle pain model has been proved to be secure [24] and all the subjects were allowed to leave until no pain felt in the neck region and no other uncomfortable feelings.

### Fluoroscopic image analysis and reposition error

**Fig. 1:**
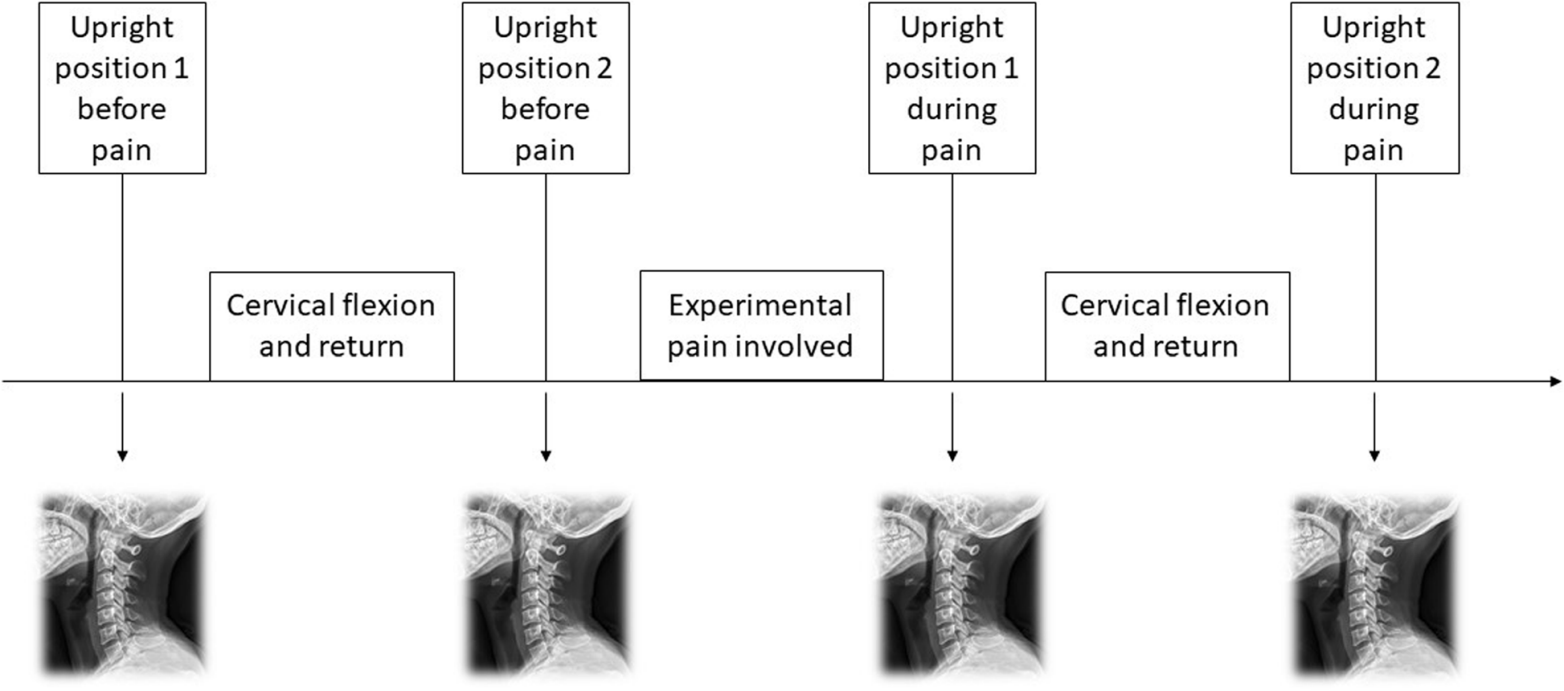
Flow chart of the experimental procedure and the four upright positions in the procedure

The subjects were asked to sit on a wood chair (same position and procedure as the previous study utilized) in the cervical upright position (upright position 1 before pain) looking straight forward at a cross on the wall (the cross was adjusted according to the height of the subjects making sure look forward straight) [3]. They were asked to return as precisely as possible to the cervical upright position (upright position 2 before pain) after full cervical flexion movement. The same was recorded during experimental multifidus muscle pain as (upright position 1 during pain) and (upright position 2 during pain). The four upright position images by fluoroscopy (Cios Alpha, Simens Healthineers, 2015, Germany) were applied for reposition error calculation. The subjects were instructed to practise several times before strictly following the experiment procedure (Fig. 1). The angles of C0/C1-C6/C7 in each upright position image were analyzed and calculated as the degree between two adjacent midplane line of each vertebrae [3, 4, 25, 29–32]. The midplane line of each vertebrae were derived from the vertebral corners as anatomical landmarks by Frobin et al. for more details and from former published studies with good reliability and reproducibility (Marking procedures on Supplementary Fig. 1) [3, 31, 33].

**Fig. 2:**
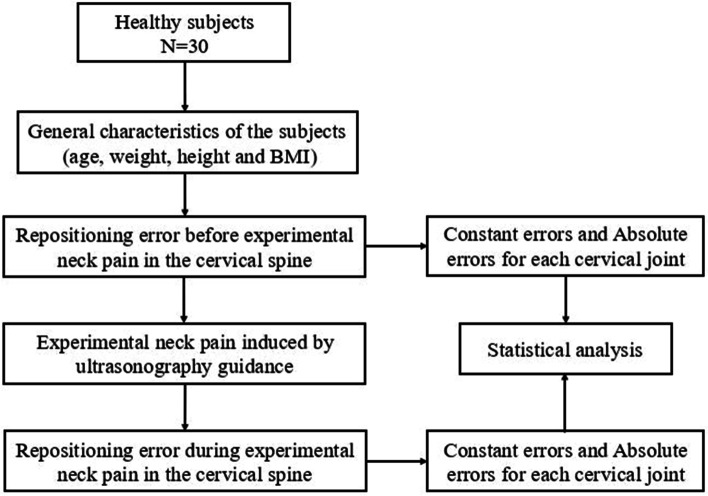
Flow-chart of the study. N: number; BMI: body mass index

The reposition errors were calculated as the difference in degrees of the cervical joint angles in upright positions (upright 1 control – upright 2 control AND upright 1 pain – upright 2 pain). The errors were calculated as constant errors (CEs) and absolute errors (AEs) in degrees. CE represents the multidirectional magnitude of under or over estimation of the target position and AE represents the only magnitude with no directions and is calculated as the absolute value of CE [20, 34–36]. The study was committed following the flow chart (Fig. 2).

Investigator XW marked all the fluoroscopic images (upright position image) three times to test the intra-rater reliability [29]. The average radiation dose for each subject was calculated to be 0.12 mSv by PCXMC [37].

### Statistics

Mean and standard deviations (SD) were presented in text while mean and standard error (SE) were presented in figures. Statistical analysis was performed in SPSS (IBM statistics version 26). Prior to the statistical analysis, all the data were tested for normal distribution by the Kolmogorov-Smirnov test and the homogeneity of variance between paired conditions was tested by Mauchly’s test.

To assess if experimental pain from multifidus show effects on the repositioning errors across the cervical joints, either constant errors or absolute errors was analysed by a two-way repeated measures analysis of variance (RM-ANOVA) with factors: Joint (C0/C1, C1/C2, C2/C3, C3/C4, C4/C5, C5/C6, C6/C7) and Time (before pain and during pain). Post hoc was performed with Bonferroni correction for multilevel comparison if it was still significant after Family wise corrections. *P* <.05 was set to be significant.

The measurement errors assessed in the subjects were presented as mean (±SD) and the test-rested reliability was tested with intra-class correlation coefficient (ICC 3,1).

## Results

The analysis included 1680 joint reposition errors by thirty subjects*seven joints*2 (control and pain) *2 (AEs and CEs). The intra-rater measurement errors and ICC of the upright position image were 0.11°±0.39° and 0.998. The measurement error was normally distributed.

### Cervical joint CEs

**Fig. 3:**
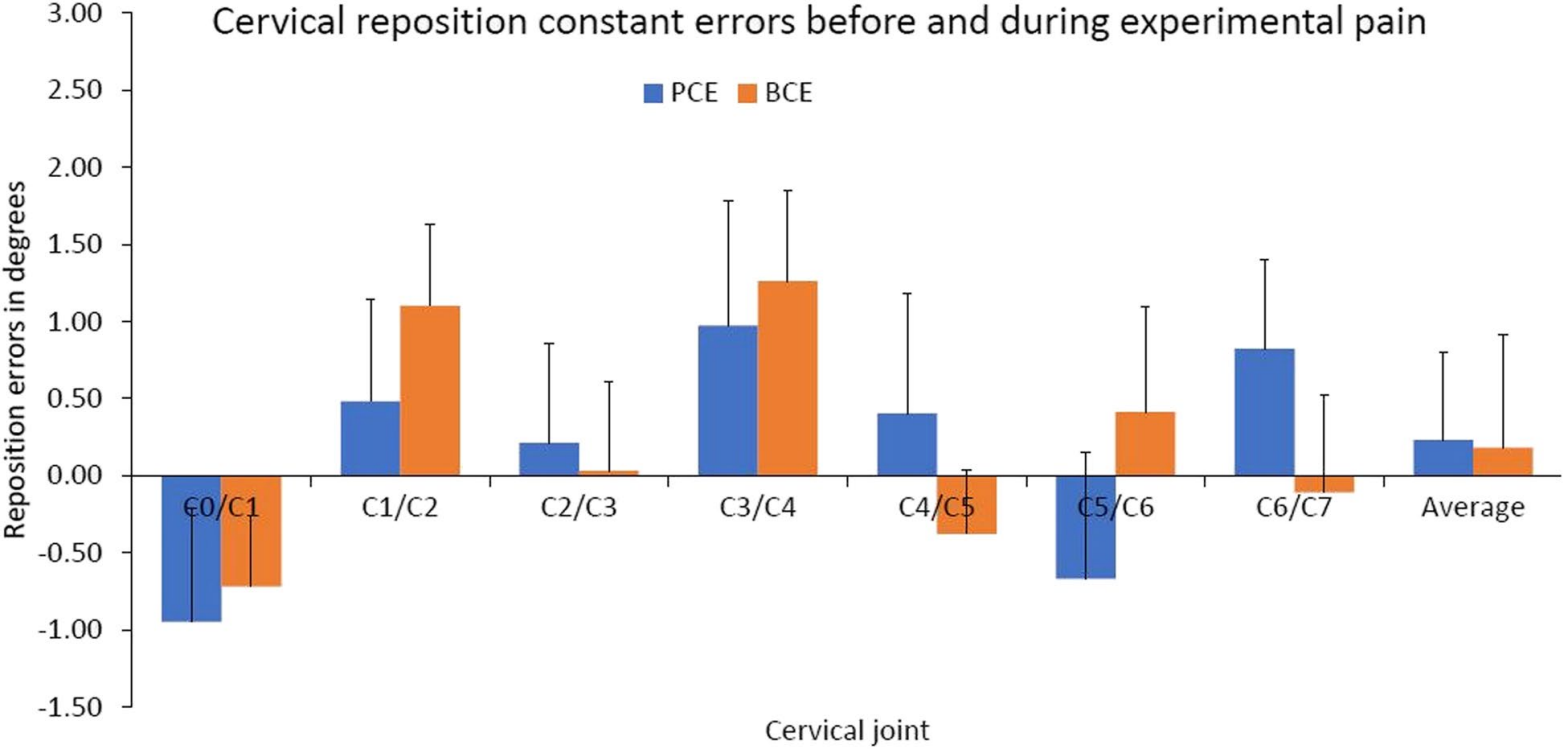
Mean (+SE) joint reposition constant error of the cervical spine before experimental pain

The average cervical joint CEs before pain and during pain were 0.18°±2.18° and 0.23°±2.22°, respectively. No main effects on either time factor, joint factor or interceptions between time and joint were detected by RM-ANOVA (Fig. 3).

### Cervical joint AEs

**Fig. 4:**
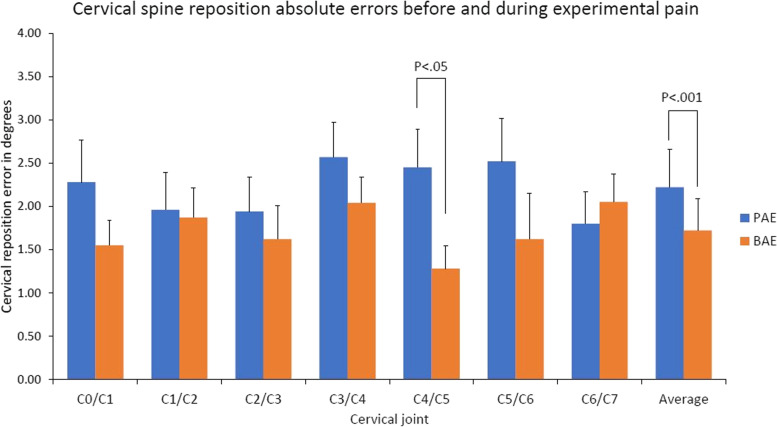
Mean (+SE) joint reposition absolute error of the cervical spine during experimental pain. The absolute error of C4/C5 joint during experimental pain was larger compared to that of C4/C5 before experimental pain (Bonferroni: *P*=0.035)

The average cervical joint AEs before and during pain were 1.30°±0.79° and 2.22°±1.72°, respectively. Main effect of time was significant before and during pain (Fig. 4, F_(1,29)_=5.879, P=0.017). Significant interaction of joint and time was found before and during multifidus muscle pain condition (Fig. 4, F_(6,174)_=281.441, *P*<0.001). Post hoc analysis revealed larger AEs of C4/C5 during pain compare to before pain (Bonferroni: *P*=0.035). Although no significant difference detected, the AEs of C3/C4 and C5/C6 (adjacent joint to C4/C5) were also larger during pain compared to before pain (Bonferroni: *P*=0.329 and *P*=0.103, respectively).

## Discussion

The cervical spine demonstrated non-significant difference in reposition errors regarding the constant errors while larger reposition errors regarding absolute errors during experimental pain compared to before experimental pain. In addition, the pain level joint and its adjacent joints indicated larger reposition errors regarding absolute errors.

### Constant errors and absolute errors

The averaged cervical spine reposition errors regarding constant errors and absolute errors before pain in this study were 0.18°±2.18° and 1.30°±0.79°, respectively. This is in line with the constant errors 0.21°±0.28° and absolute errors 2.36°±0.19° from former study while the variation was presented by standard error of measurement (SEM) [3]. Additionally, the constant errors before pain in this study demonstrated similar tendency with the former study that the upper and lower cervical joints showed reversed direction of repositioning compared to the middle cervical joints [3].

### Experimental pain and reposition errors

With regard to the absolute errors, the results confirmed the hypothesis in this study that the reposition errors were larger during experimental pain compared to before experimental pain, and it also confirmed the second hypothesis that the cervical joints adjacent to the pain level showed larger reposition errors compared to joints distant to the pain site. However, the cervical spine demonstrated non-significantly different reposition errors during pain compared to before pain regarding constant errors. This may be explained that the cervical spine could self-balance the upright position even during neck pain while the pain did not influence the structure and function (as the cervical spine can flex to the end and return) of the cervical spine [25, 30]. The absolute errors showed that the cervical spine indicated larger reposition errors during pain conditions, which is in accordance with former studies that neck pain results in impaired proprioception reflected by increased reposition errors [21, 38–43]. However, the former studies investigating the neck pain reposition errors considered the neck as a whole unit by wearing a CROM (cervical range of motion) device, which could not investigate the reposition errors inside the cervical spine (e.g. specific joint) [44].

The current study is the first to investigate the reposition errors of specific cervical joints during pain, that the joints adjacent to the pain level showed impaired proprioception reflected by larger reposition errors. This could be explained by the deep cervical muscles anatomically distribute to the local cervical joints rather than distant joints [6, 25].

Cervical experimental muscle pain model was frequently applied to investigate the origin and depth of pain effects on cervical spine function and disorders [6]. In this study, the experimental multifidus pain presented the deep and local cervical muscle pain effects on the cervical spine proprioception, which mimics the local pre-clinical neck pain effects on the cervical spine proprioception reflected by increased reposition errors. This may imply to the clinicians that the larger cervical joint reposition errors indicating local injury existed.

### Clinical and scientific implications

This study firstly demonstrated the cervical joint reposition errors in healthy subjects induced experimental deep cervical muscle pain mimicking the pre-clinical neck pain. The errors further showed that the cervical joints adjacent to the pain level indicated larger errors compared to the joints distant to the pain level. This may be a possible clue for clinicians for identifying the origin and depth of injury when specific joints showed impaired proprioception reflected by larger reposition errors. More importantly, the possibilities need to be further investigated in other cervical spine disorders such as cervical radiculopathy, whiplash, and trauma conditions to test the reliability and reproducibility before applied.

Cervical joint reposition errors were firstly examined during experimental pain in this study, which opens the possibilities of investigating the specific joint proprioception for precisely identifying the origin and depth of neck pain in future studies. In addition, the results in this study, to some degree, explains the conflict results of neck proprioception studies investigating cervical spine as a whole unit rather than multi-unit structure is a probable confounding in former studies [3].

### Future perspectives

The specific cervical joint reposition error has rarely been investigated as many previous studies considering the cervical spine as a whole unit rather than multi-unit structure. The repositioning accuracy reduction of an individual joint may reflect the problem adjacent to the cervical level. The specific joint reposition error can also be applied as a potential parameter for evaluating the treatment by increasing or decreasing to study the disease. While the underlying mechanism of the specific joint repositioning is unclear as it compromises local and distal muscles around it. In addition, investigations of cervical joint reposition error in different conditions such as neck pain, chronic pain, cervical radiculopathy and degenerative diseases are needed.

### Study limitations

This study has several limitations. Firstly, the measurement error was a large source of errors in this study. However, the reproducibility and repeatability of the marking procedure have been validated with good reliability and low average marking error [31]. Secondly, the multifidus experimental pain was induced in the right one while this may lead to the asymmetry pain in the cervical spine, and the asymmetry pain may distort the cervical spine especially when moving occurs. The reposition errors based on the marking image analysis may be influenced by the distortion due to asymmetry pain in multifidus. Thirdly, the cervical spine reposition errors after movement were only investigated after flexion movement in this study, which may influence the experimental pain effects on neck proprioception because the sagittal movement includes extension and flexion. This is due to the size of the fluoroscopic screen restricting the movement included and flexion can better reflect the proprioception of the cervical spine. Fourthly, the age (less than 40 years in average) of the subjects included may not entirely mimic the renal neck pain conditions after experimental neck pain induced and this may influence the results in this study as most neck pain occurs in age 45-55 years [45].

## Conclusion

This study firstly investigated the cervical joint reposition errors in healthy subjects induced experimental neck pain and further found the joints adjacent to the pain level showed larger reposition errors compared to the distant joints regarding absolute errors. For clinicians, this may imply that the larger reposition errors in specific cervical joint indicate that possible injury or pain existed adjacent to the joints.

## Supplementary Information

**Additional file 1.**

## Data Availability

The datasets generated and/or analysed during the current study are not publicly available due to limitations of hospital ethical approval involving the patient data and anonymity but are available from the first author or corresponding author on reasonable request.
