# Supplementary figures and images for: Effects of experimental pain on the cervical spine reposition errors
Source: BMC Musculoskelet Disord. 2022 Mar 17;23:259. doi: 10.1186/s12891-022-05170-7 (PMC8932173; doi:10.1186/s12891-022-05170-7)

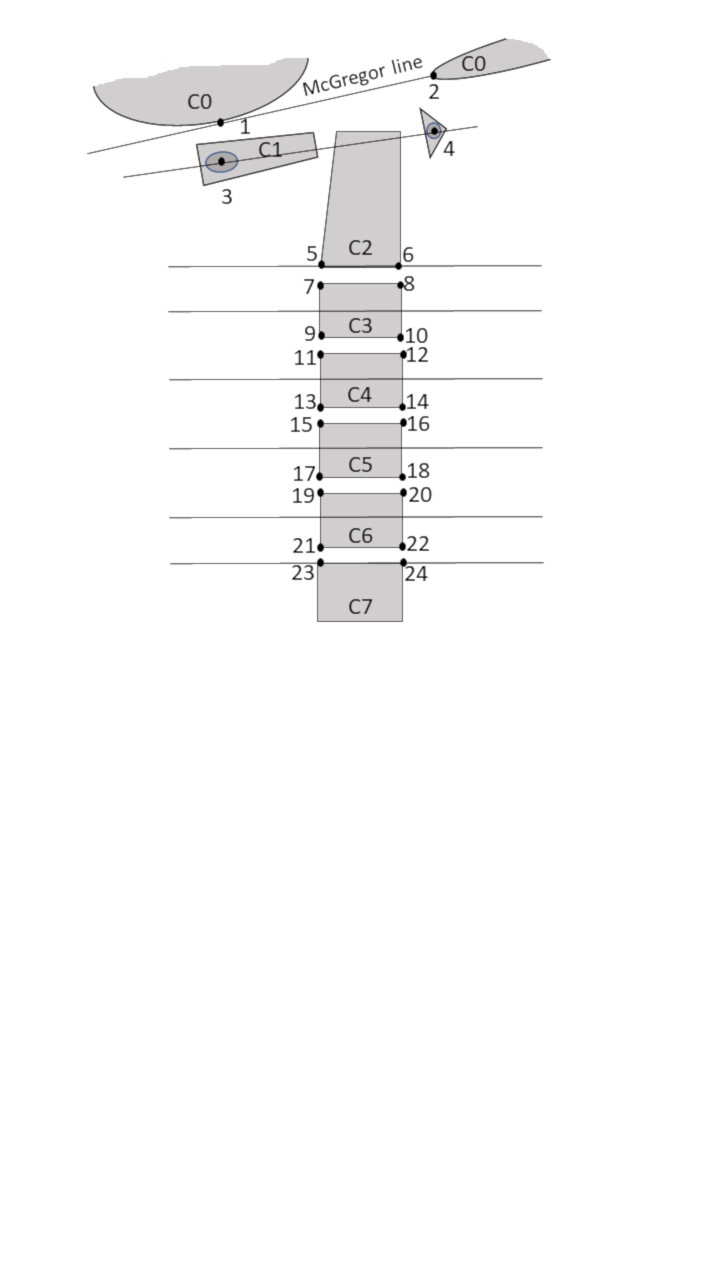

Supplement: Supplementary file 1 — Additional file 1. [file 12891_2022_5170_MOESM1_ESM.tif]
